# Relation of intraoperative temperature to postoperative mortality in open colon surgery—an analysis of two randomized controlled trials

**DOI:** 10.1007/s00384-015-2467-4

**Published:** 2015-12-23

**Authors:** J. Frey, M. Holm, M. Janson, M. Egenvall, J. van der Linden

**Affiliations:** Department of Cardiothoracic Surgery and Anesthesiology, Karolinska Institute, Karolinska University Hospital, Stockholm, Sweden; Department of Molecular Medicine and Surgery, Karolinska Institute, Karolinska University Hospital, Stockholm, Sweden; Department of Clinical Sciences, Interventions and Technology; Division of Surgery, Karolinska Institute; Karolinska University Hospital, Stockholm, Sweden

**Keywords:** Carbon dioxide, Colon cancer, Survival, Surgery, Temperature

## Abstract

**Introduction:**

The open surgical wound is exposed to cold and dry ambient air resulting in heat loss mainly through radiation and convection. This cools the wound and promotes local vasoconstriction and hypoxia. Carbon dioxide (CO_2_) and water vapor are greenhouse gases with a warming effect. The aim was to evaluate if warm humidified CO_2_ insufflated in surgical wound can affect long-term overall mortality

**Methods:**

This is a retrospective study of two clinical trials, where patients were randomized to warm humidified CO_2_ (*n* = 80) or not (*n* = 78). All patients underwent elective major open colon surgery. Patients in the treatment group received insufflation of warm humidified CO_2_ into the open wound cavity via a gas diffuser to create a local atmosphere of 100 % CO_2_. Temperature in the wound cavity was measured with a heat-sensitive infrared camera. Core temperature was measured at the tympanic membrane. Median follow-up was 70.9 months.

**Results:**

A multivariate analysis adjusted for age (*p* = 0.001) and cancer (*p* = 0.165) showed that the larger the temperature difference between final core temperature and wound edge temperature, the lower the overall survival rate (*p* = 0.050). Patients receiving insufflation of warm humidified CO_2_ had a tendency to a better overall survival compared with control patients (*p* = 0.508). End-of-operation wound edge temperature was negatively associated with mortality (OR = 0.80, 95 % CI = 0.68-0.95, p = 0.011), whereas mortality was positively associated with age (10-year increase, OR = 1.78, 95 % CI = 1.37-2.33, p < 0.001) and cancer (OR = 8.1, 95 % CI = 1.95–33.7, p = 0.004).

**Conclusions:**

A small end-of-operation temperature difference between final core and wound edge temperature was positively associated with patient survival in open colon surgery.

## Introduction

Open colorectal surgery under general anesthesia almost always results in intraoperative hypothermia. This is due to anesthesia-induced thermoregulatory inhibition combined with exposure to a cold operating room environment [1]. Heat loss through convection and radiation accounts for the majority of the total perioperative heat loss. A large open surgical wound will amplify evaporation and radiation from the exposed surfaces of the internal organs. Hypothermia is traditionally defined as a core temperature of <36.0 °C [2], and perioperative core hypothermia increases the risk of surgical wound infection [3–5], as a consequence of decreased local tissue blood flow and tissue oxygenation [6, 7]. Also, it impairs immune function [8, 9], increases perioperative bleeding and transfusion requirements [10], enhances the incidence of postoperative shivering and morbid cardiac events [11], and it prolongs hospital stay and increases costs. Current guidelines [2, 12–14] therefore recommend active counter measures to maintain normothermia, including passive insulation and, active transfer of heat to the body with resistive-heating or forced-air warming blankets, as well as fluid warming systems. However, more than one third of general surgery patients undergoing open abdominal operations have been reported to be hypothermic on arrival in the postoperative care unit [14, 15].

Warming the open surgical wound by local insufflation of warmed humidified carbon dioxide (CO_2_) during colorectal surgery has been shown to significantly increase both core and wound temperatures in two randomized clinical trials [16, 17]. The creation of a CO_2_-saturated atmosphere within the surgical wound cavity offers unique possibilities, because CO_2_ acts like a greenhouse gas that will trap heat radiating from the organ surfaces and decrease evaporative heat loss and convection resulting from the operating room ventilation [18, 19]. Furthermore, locally applied CO_2_ is known to cause vasodilatation of micro vessels in the skin and muscular arterioles [20], leading to increased local tissue perfusion in the surgical wound.

The aim of the present study was to investigate whether increased wound and core temperatures during major open colorectal surgery induced by local insufflation of warm humidified CO_2_ is related to long-term overall morbidity and mortality.

## Methods

This study is a post hoc retrospective single-center study of two clinical trials where patients were randomized to a warm humidified CO_2_ group (*n* = 80) or a control group (*n* = 78). All included patients underwent elective major open colon surgery between March 2007 and November 2013 at Karolinska University Hospital. For this type of study, formal consent is not required. Postoperative morbidity and mortality were obtained May 2015, from the hospital’s medical records that are linked to the national Swedish database on mortality, the Total Population Registry (Approved by the Regional Ethical Committee in Stockholm, 2015/1063-32). None of the patients were lost to follow-up. Patients randomized to the treatment group received insufflation of warm humidified CO_2_ into the open wound cavity via a humidification system and a gas diffuser (Carbon Vita®, Cardia Innovation AB, Stockholm, Sweden). In the first trial [17], a noncommercial system that delivered humidified CO_2_ at 93 % relative humidity and 30 °C (*n* = 80) was used, whereas in the second trial [16], a commercial system delivered 100 % relative humidity and 37 °C (*n* = 78) to the surgical wound (HumiGard™, Fisher & Paykel HealthCare Ltd, Auckland, New Zealand). All but 10 patients (6 %) received a thoracic epidural blockade in addition to general anesthesia. The temperature in the wound cavity was measured every 10 min with a heat-sensitive infrared camera (ThermaCAM™ B2, FLIR Systems AB, Danderyd, Sweden). Core temperature was measured in degrees Celsius at the tympanic membrane every 30th minute by a thermometer (CORE-CHECK™ Tympanic Thermometer System, Cardinal Health, Dublin, OH) from the time that the patient was anesthetized until the end of surgery. Further details regarding the trials were previously published [16, 17]. In addition to the exclusion criteria described [16, 17], patients who underwent colostomy surgery were excluded since the focus was on major colon surgery.

### Statistical analyses

Data are presented as numbers and percentages. Survival in the CO_2_ group and the treatment group, as well as final core temperature ≥36.0 °C, respectively, was analyzed using Kaplan-Meier curves. To identify variables associated with mortality, univariate Cox regression analysis was performed. The relationship between core and wound edge temperature differences at the end of surgery, and mortality, was analyzed using the Cox proportional hazards model adjusted for age and cancer. The *p* values for the differences in patient characteristics were obtained by chi-square or *t* tests. SPSS software (version 22, SPSS Inc, Chicago, Ill) was used for statistical analyses. All tests were two-sided. Statistical significance was accepted for *p* values ≤0.05.

## Results

The total study population comprised of 91 men and 67 females with a median age of 63 years. Median follow-up was 70.9 months, and no patients were lost to follow-up. Preoperative patient characteristics did not differ significantly between the treatment groups Table 1.

**Table 1:** Demographic characteristics of the study cohort including comparisons between patients with and without humidified warmed CO_2_

Characteristic	Randomization		
	Humid warm CO_2_	Controls	*p* value
	(*n* = 80)	(*n* = 78)	
Clinical parameters			
Age, years	62.9 ± 14.0	63.4 ± 17.7	0.833
Male gender	46 (57.5 %)	45 (57.7 %)	0.980
BMI, kg/m^2^	25.5 ± 4.5	25.3 ± 4.4	0.787
Colon/rectal cancer	59 (73.8 %)	58 (74.4 %)	0.930
Primary surgery	72 (90.0 %)	64 (82.1 %)	0.149

^a^Data are presented as mean ± SD for quantitative variables, and as No. (%) for qualitative variables

*BMI* body mass index

All temperatures at the end of surgery as well as the temperature differences between core and wound were significantly higher in the CO_2_ group. Mean operating time was 218 min in both groups, and all remaining end points tended to be in favor of the CO_2_ group (Table 2; peri- and postoperative end points).

**Table 2:** End points

End point	Randomization		
	Humid warm CO_2_	Controls	*p* value
	(*n* = 80)	(*n* = 78)	
Operation duration (min)	218.0 ± 97.2	218.0 ± 94.2	1.0
Anesthesia time (min)	297.3 ± 108.9	302.5 ± 104.9	0.775
Intraoperative bleeding	473.7 ± 613.1	468.3 ± 537.3	0.954
Mean core temperature	36.2 ± 0.6	35.9 ± 0.5	0.005
Mean core temperature ≥36.0 °C	51 (64.6 %)	32 (42.7 %)	0.006
Mean wound edge temperature	29.8 ± 1.2	28.5 ± 1.1	<0.001
Mean wound area temperature	31.0 ± 1.2	29.7 ± 1.1	<0.001
Final core temperature	36.5 ± 0.6	36.1 ± 0.6	<0.001
Final core temperature ≥36.0 °C	66 (82.5 %)	49 (65.3 %)	0.015
Final wound edge temperature	29.7 ± 1.9	28.5 ± 1.7	<0.001
Final wound area temperature	31.2 ± 2.0	30.1 ± 1.9	0.001
Mean core—mean wound edge temp	6.4 ± 1.1	7.4 ± 1.1	<0.001
Mean core—mean wound area temp	5.2 ± 1.1	6.2 ± 1.2	<0.001
Final core—last wound edge temp	6.8 ± 1.8	7.7 ± 1.7	0.006
Final core—last wound area temp	5.3 ± 1.9	6.0 ± 1.9	0.023
Wound rupture	1 (1.3 %)	3 (3.8 %)	0.364
Re-operation	7 (8.8 %)	7 (9.0 %)	0.960
Surgical site infection <30 days	13 (16.3 %)	13 (16.7 %)	0.944
Mortality	19 (23.8 %)	22 (28.2 %)	0.519
Readmission <30 days	12 (15.0 %)	13 (16.7 %)	0.774
PRBC transfused (units)	0 [0–13]	0 [0–9]	0.738
Plasma transfusion	0 [0–12]	0 [0–5]	0.600
Platelet transfusion	0 [0–4]	0 [0–2]	0.992

^a^Data are presented as No. (%), mean ± standard deviation, or as median [range]

Of the 158 patients, 117 (74 %) patients underwent open colon/rectal cancer surgery, with the remainder operated on for inflammatory bowel disease involving the colon. Forty-one (26 %) died during the complete follow-up period including 3 patients (2 %) who died within 30 days of the operation. Primary causes of death (disease or condition directly leading to death) within 30 days were cardiovascular (*n* = 3), whereas the primary causes of death after 30 days were cancer (*n* = 20), cardiovascular (*n* = 7), ileus (*n* = 1), renal insufficiency (*n* = 1), sepsis (*n* = 1), and unknown (*n* = 8).

**Fig. 1:**
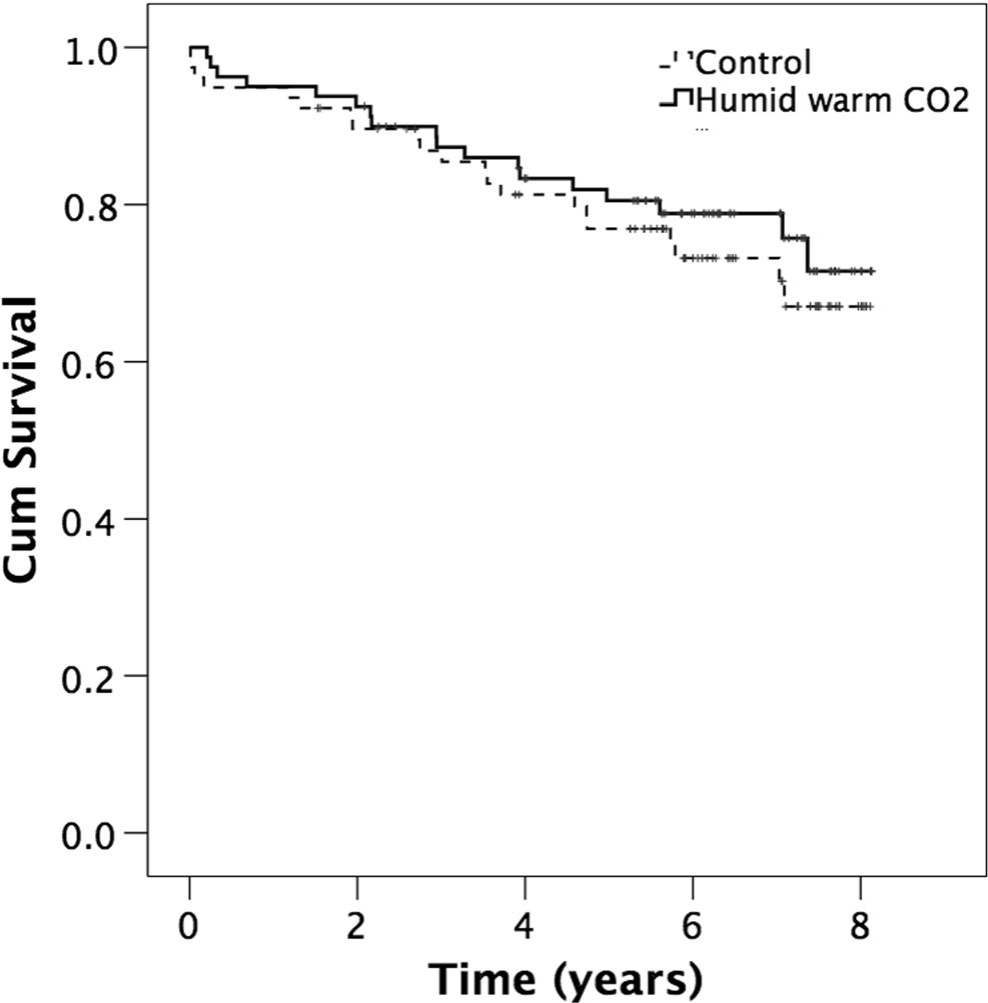
Cumulative survival in the CO_2_ and control group in all subjects after major open colon surgery (log rank *p* = 0.508). *Small vertical lines* represent end of follow-up

**Fig. 2:**
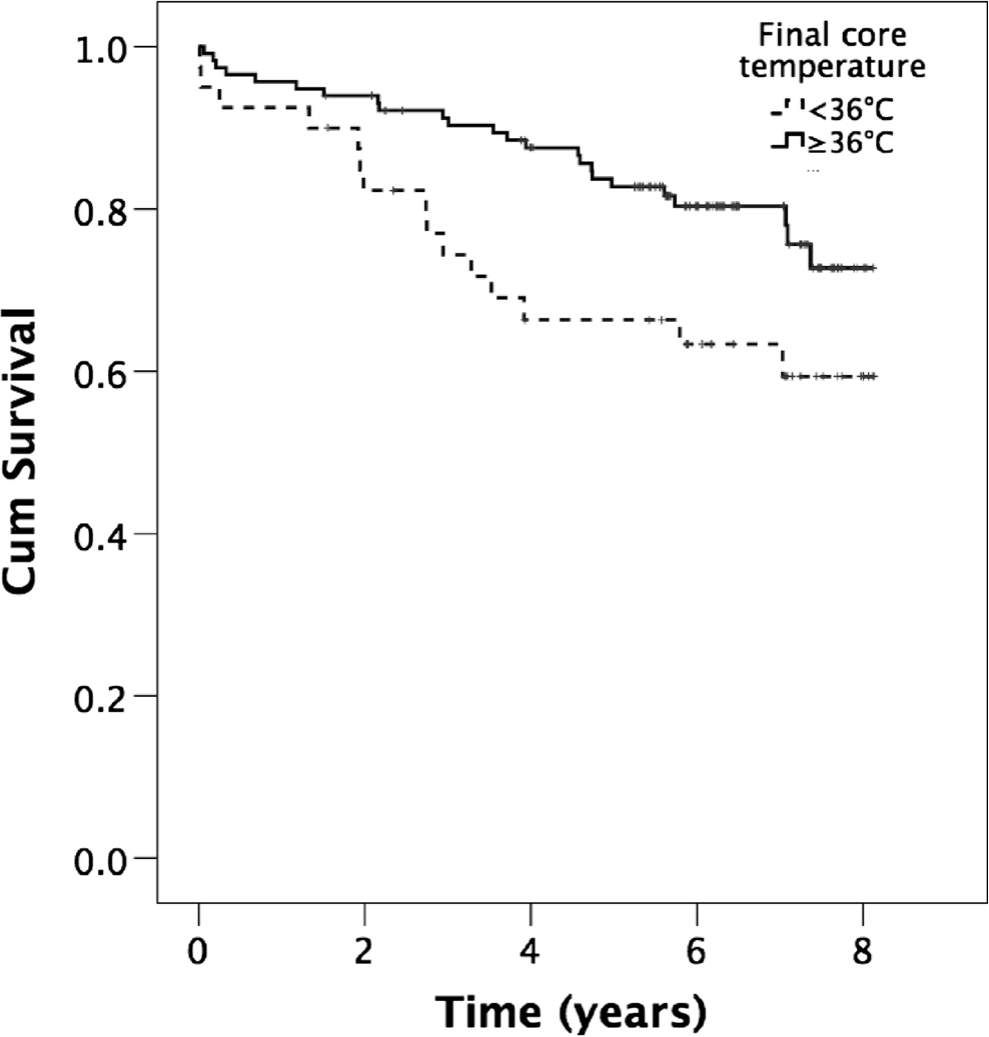
Cumulative survival in patients with a core temperature ≥36.0 and <36.0 °C at end of surgery in all subjects after major open colon surgery (log rank *p* = 0.035)

Patients receiving insufflation of warm humidified CO_2_ had a tendency to a better overall survival compared with control patients (*p* = 0.508). Figure 1 depicts the survival in the CO_2_ group and the treatment group in all subjects. Patients with a core temperature ≥36.0 °C at the end of surgery exhibited a better overall survival compared with those with core temperature <36.0 °C at the end of surgery (OR 0.5, 95 % CI 0.26-096, *p* = 0.035), Fig. 2.

Overall univariate mortality predictions for all patients during elective major open colon cancer surgery are shown in Table 3. As expected, age and cancer showed a strong association with mortality (*p* = <0.001 and *p* = 0.004, respectively). Moreover, a final core temperature ≥36.0 °C (*p* = 0.035) and a higher final wound edge temperature (*p* = 0.011) were associated with lower mortality, whereas a smaller difference between final core and final wound edge temperature (*p* = 0.017) improved survival. A multivariate analysis (Table 3) adjusted for age (*p* = 0.001) and cancer (*p* = 0.165) showed that the temperature difference between final core and final wound edge temperature was associated with a better overall survival (*p* = 0.050).

**Table 3:** Cox analysis for the prediction of mortality

Variable	Univariate analysis		Multivariate analysis	
	HR (95 % CI)	*p* value	HR (95 % CI)	*p* value
Mean core—mean wound edge temp	1.24 (0.96–1.59)	0.097		
Mean core—mean wound area temp	1.15 (0.90–1.48)	0.256		
Final core—final wound edge temp	1.24 (1.04–1.47)	0.017	1.20 (1.00-1.44)	0.050
Final core—final wound area temp	1.13 (0.97–1.32)	0.125		
Age (10-year increase)	1.78 (1.37–2.33)	<0.001	1.05 (1.02–1.08)	0.001
Cancer	8.1 (1.95–33.7)	0.004	2.92 (0.64–13.3)	0.165
Final core temperature ≥36 °C	0.50 (0.26–0.96)	0.035		
Mean core temperature	0.95 (0.54–1.69)	0.869		
Mean core temperature ≥36 °C	0.93 (0.50–1.75)	0.821		
Mean wound area temperature	0.87 (0.68–1.10)	0.242		
Mean wound edge temperature	0.81 (0.63–1.03)	0.089		
Final core temperature	0.86 (0.51–1.43)	0.551		
Humidified warmed CO_2_	0.80 (0.43–1.50)	0.490		
Final wound area temperature	0.88 (0.76–1.02)	0.095		
Final wound edge temperature	0.80 (0.68–0.95)	0.011		

## Discussion

This is a hypothesis-generating, retrospective single-center study following two smaller randomized trials. This work has shown that long-term mortality is associated with core and wound edge temperatures at the end of major open colorectal surgery as well as to age and cancer diagnosis. The difference between core and wound edge temperature at end of surgery significantly influenced mortality in a multivariate model, when controlling for age and cancer diagnosis. Insufflation of warmed humidified CO_2_ in the open surgical wound increased final core and wound temperatures during surgery but did not significantly affect mortality.

The potential ability of increased wound temperature to improve long-term survival after major open colon surgery can be attributed to at least three different mechanisms.

First, perioperative hypothermia has been demonstrated to lead to increased cardiac demand and, subsequently, increased risk of cardiac morbidity [21]. Patients who survived a postoperative cardiac event continued to be at a considerable risk of cardiac death, with a hazard ratio of 18 (95 % CI, 6–57) in the first 6 month after discharge. In patients with cardiac risk factors who are undergoing noncardiac surgery, the perioperative maintenance of normothermia is associated with a reduced incidence of morbid cardiac events and ventricular tachycardia [11]. These numbers are consistent with our findings that patients with a core temperature ≥36.0 °C at the end of surgery exhibited a significantly better overall survival compared with those with core temperature <36.0 °C at the end of surgery. Also, the treatment group with insufflation of warm humidified CO_2_ tended to have a better longtime survival, although this did not reach significance, possibly due to a type II error.

Second, insufflation of warm humidified CO_2_ in the open surgical wound increased core and wound temperatures and decreased the difference between core and wound temperatures. These changes may indicate a better perfusion and a better oxygenation of the open surgical wound, where wound edge temperature is a more sensitive indicator of wound tissue perfusion than wound area, since the latter temperature is influenced by all exposed internal tissues. A recently published rat model showed that insufflation of warm humidified CO_2_ into the abdominal cavity during open abdominal surgery caused a rapid increase in wound tissue oxygen tension [22]. The humidification and warming to physiological temperature of the insufflated CO_2_ decrease desiccation from the open wound and increase overall wound temperature thereby improving general wound perfusion and oxygenation.

Third, preventing desiccation of the exposed peritoneal mesothelium by insufflation of warm humidified CO_2_ has been shown to reduce intraperitoneal tumor dissemination in animal models, a finding which is consistent with maintaining the physiological integrity of the mesothelium as an intact barrier to tumor infiltration [23, 24]. The peritoneum was the sole site of metastasis in >50 % of patients with metastatic disease [25], and such metastasis remains fatal [26]. This could have impacted the long-term survival in our study.

Potential limitations are that this was a retrospective post hoc study of postoperative morbidity and mortality, although patients had been randomized before surgery. Potentially relevant data are missing in the records such as prospective evaluation of wound infections. Moreover, the study is relatively small with a concurrent probability of type II errors. Also, two different heating systems were used in the study.

The strength of the study is that the long-term effects of intraoperative wound area and wound edge temperatures have to our knowledge not been studied before. Importantly, patients were warmed with standard warming measures according to the NICE guidelines [2]. In addition, 94 % of the patients received epidural analgesia together with general anesthesia, and this combination has been shown to increase long-term survival after colon surgery in a retrospective study [27], which could be due to a reduction in neuroendocrine response and attenuated immunosuppression. Furthermore, none of our patients were lost to follow-up.

In conclusion, our study shows that insufflation of warm humidified CO_2_ into the open wound significantly increases wound and core temperatures. Normothermia at end of surgery as well as a small end-of-operation temperature difference between final core and wound edge temperature was significantly associated with better patient survival in open colon surgery. Moreover, patients with a core temperature ≥36.0 °C at end of surgery exhibited a better overall survival compared with those with core temperature <36.0 °C.
